# Maternal Malaria and Perinatal HIV Transmission, Western Kenya^1^^,^^2^

**DOI:** 10.3201/eid1004.030303

**Published:** 2004-04

**Authors:** John G. Ayisi, Anna M. van Eijk, Robert D. Newman, Feiko O. ter Kuile, Ya Ping Shi, Chunfu Yang, Margarette S. Kolczak, Juliana A. Otieno, Ambrose O. Misore, Piet A. Kager, Renu B. Lal, Richard W. Steketee, Bernard L. Nahlen

**Affiliations:** * Centre for Vector Biology and Control Research, Kenya Medical Research Institute, Kisumu, Kenya; † University of Amsterdam, Amsterdam, The Netherlands; ‡ Centers for Disease Control and Prevention, Atlanta, Georgia, USA; § Ministry of Health, Kisumu, Kenya; ¶ World Health Organization, Geneva, Switzerland

**Keywords:** malaria, HIV, pregnancy, vertical disease transmission, placenta, risk factors, Kenya, Africa

## Abstract

To determine whether maternal placental malaria is associated with an increased risk for perinatal mother-to-child HIV transmission (MTCT), we studied HIV-positive women in western Kenya. We enrolled 512 mother-infant pairs; 128 (25.0%) women had malaria, and 102 (19.9%) infants acquired HIV perinatally. Log_10_ HIV viral load and episiotomy or perineal tear were associated with increased perinatal HIV transmission, whereas low-density malaria (<10,000 parasites/μL) was associated with reduced risk (adjusted relative risk [ARR] 0.4). Among women dually infected with malaria and HIV, high-density malaria (>10,000 parasites/μL) was associated with increased risk for perinatal MTCT (ARR 2.0), compared to low-density malaria. The interaction between placental malaria and MTCT appears to be variable and complex: placental malaria that is controlled at low density may cause an increase in broad-based immune responses that protect against MTCT; uncontrolled, high-density malaria may simultaneously disrupt placental architecture and generate substantial antigen stimulus to HIV replication and increase risk for MTCT.

Malaria during pregnancy is a serious problem in sub-Saharan Africa, affecting an estimated 24 million pregnant women; malaria prevalence may exceed 50% among primigravid and secundigravid women in malaria-endemic areas (*1*). During the past 2 decades, HIV and AIDS have emerged as major problems in many malaria-endemic areas of sub-Saharan Africa, where an estimated 28 million persons are infected with HIV (*2*). Sub-Saharan Africa accounts for more than two thirds of the world’s 40 million HIV-infected persons and 80% of the world’s HIV-infected women, with HIV prevalence rates sometimes exceeding 40% among pregnant women (*3*). Without intervention, as many as 30%–45% of pregnant women infected with HIV will pass the virus to their children through mother-to-child transmission (MTCT) (*4*), of which 15%–30% is intrauterine or intrapartum.

Given the wide overlap between areas where HIV and malaria are each prevalent, the epidemic of HIV/AIDS in areas where *Plasmodium falciparum* is endemic has generated concern about potential interactions between the two infections, especially in sub-Saharan Africa (*5*–*9*). Studies have shown increased HIV replication both in blood mononuclear cells exposed to malaria antigens in vitro (*10*) and in transgenic mice infected with *P. chabaudi* (*11*). Proviral loads are also higher among HIV-infected persons with clinical malaria compared to those without malaria; these levels remain high for at least 4 weeks after treatment (*12*). Although malaria may increase viral replication in the short term, the concern that the malaria-associated increase in viral replication may accelerate HIV disease progression has not been proven (*13*).

Studies among pregnant women in sub-Saharan Africa have provided the first evidence of an important public health problem arising from the interaction of HIV and malaria. HIV infection appears to impair malarial immunity among pregnant women, as pregnant women infected with HIV demonstrate more frequent and higher density parasitemia than pregnant women not infected with HIV (*5*–*7*,*14*). More recent case-control (*15*) and longitudinal (*16*,*17*) studies on the clinical pattern of malaria in HIV-infected, nonpregnant women have shown HIV infection to be associated with an increased frequency of clinical malaria and parasitemia, particularly among persons with advanced HIV disease. Data from Malawi (*18*) have suggested that infants exposed to both placental malaria and maternal HIV infection have an increased risk for postneonatal death three- to eightfold higher than infants born to mothers with either infection alone. We examined the relationship between placental malaria and vertical HIV transmission in an area of western Kenya with high prevalence of malaria and HIV infection.

## Participants and Methods

### Study Site

This study was conducted at Nyanza Provincial General Hospital (NPGH), a large publicly funded hospital in Kisumu (population 300,000) in western Kenya. Malaria transmission within Kisumu is perennial, and *P. falciparum* is the predominant species, accounting for 98% of malaria cases (*19*). Chloroquine resistance is prevalent in the area: 75%–80% of *P. falciparum* strains show a RII/RIII resistance pattern (*20*). The prevalence of HIV infection among pregnant women is approximately 25% (*6*,*21*).

### Recruitment and Follow-up

Pregnant women were enrolled from June 1996 through May 2000. Screening procedures have been described previously (*21*). Briefly, women at the antenatal clinic were eligible for participation if they had an uncomplicated singleton pregnancy of >32 weeks’ gestation (based on the fundal height estimation), resided within the Kisumu municipality, and had no known underlying chronic illness. Following informed consent, a structured questionnaire was administered in the local language (*Dholuo* or *Kiswahili*) to obtain information on sociodemographic, health, and obstetric factors.

A trained HIV counselor then counseled each woman, and a posttest counseling appointment was made. A blood sample was taken for HIV antibody testing, hemoglobin level, and malaria thick blood film. All screened women were encouraged to deliver at NPGH. In addition, nonscreened women who delivered at NGPH were eligible for participation if they met study inclusion criteria. Routine use of zidovudine or nevirapine was not the Kenyan Ministry of Health policy during the study period, and these drugs were not available in Ministry of Health facilities.

At delivery, information was collected on mode and outcome of delivery and any illness and treatment in the previous 2 weeks. Within 24 hours of birth, infants were weighed (±1 g) on an electronic balance (Ohaus, Florham Park, NJ), and gestational age was assessed by using the Ballard method (*22*). All live, singleton, vaginally delivered infants of HIV-positive mothers were eligible for study. Infants were seen monthly until 12 months of age.

Informed consent was obtained from all women before they were enrolled in this study. Human subjects guidelines of the Centers for Disease Control and Prevention (CDC) and the Kenya Medical Research Institute ethical review committee were strictly followed. Mothers of enrolled infants signed an additional informed consent form for infant participation.

### Ethical Review

The study protocol was approved in 1995 by the institutional review boards of the Kenya Medical Research Institute; CDC, Atlanta, Georgia, USA; and the Academic Medical Center (AMC), University of Amsterdam, Amsterdam, The Netherlands, and was reviewed annually by the participating institutions. This study occurred during a changing environment in preventing perinatal HIV transmission, with results of studies in other countries demonstrating the benefits of short-course AZT use (*23*,*24*) or nevirapine use (*25*) in late pregnancy and perinatally to reduce the risk for MTCT. Similarly, studies of malaria prevention in pregnancy during the study interval were demonstrating the benefit of intermittent antimalarial treatment to prevent maternal anemia and low birth weight (*6*,*26*). The participating institutions and the Kenyan Ministry of Health were engaged in discussions about these findings regarding the ethical considerations for this investigation. The study continued with full ethical approvals during these discussions. Investigators and institutions have supported the transition to a system that supports providing intermittent preventive antimalarial therapy during pregnancy, according to the adopted Kenyan national policy. The MTCT prevention program now offers HIV counseling and testing, community education, and antiretroviral drugs to prevent MTCT in the study setting (*4*).

### Blood Sampling and Laboratory Procedures

At delivery, maternal peripheral and placental thick blood films were prepared, stained with 10% Giemsa, and examined under oil immersion for malaria parasites. Placental blood was obtained by cutting into the maternal side of the placenta and placing collected blood on a slide. A thick film was considered negative if 100 microscopic fields showed no parasites. Malaria parasites and leukocytes were counted in the same fields until 300 leukocytes were counted. Parasite densities were estimated by using an assumed count of 8,000 leukocytes/μL blood.

Blood samples were collected into EDTA tubes. At delivery, blood was collected from mothers of enrolled infants to assess viral load, whether syphilis was present, and hemoglobin level. One month postpartum, maternal venous blood was collected to determine counts of CD4- and CD8-positive T-lymphocytes (CD4+ and CD8+). Capillary blood was collected from infants by a heel prick on the day of delivery and then monthly thereafter for HIV testing. Plasma was separated from blood samples and stored at –70°C.

Infant HIV testing was done by polymerase chain reaction (PCR) of proviral DNA extracted from peripheral blood mononuclear cells (*27*). HIV testing of pregnant women used two rapid test methods: an initial Serostrip HIV-1/2 (Saliva Diagnostic Systems Pte Ltd, Singapore) and a confirmatory Capillus HIV-1/HIV-2 (Cambridge Diagnostics, Wicklow, Ireland) on all Serostrip-positive samples. Sequential testing of samples using both methods has a high sensitivity and specificity (Richard W. Steketee, pers. comm.). Western blot was performed on discordant samples.

Maternal CD4+ and CD8+ counts were assessed by using commercial, dual-label monoclonal antibodies (Becton-Dickinson Immunocytometry, San Jose, CA) and standard fluorescent-activated cell sorting (FACScan, Becton-Dickinson) analysis following whole-blood lysis (*28*). Maternal HIV-1 viral load was determined by the Roche Amplicor HIV-1 monitor test version 1.0 (Roche Diagnostics, Indianapolis, IN), with a quantification limit of 400 viral copies per milliliter.

Syphilis antibodies were detected by venereal disease research laboratory (VDRL) slide test (EUROTEX-VDRL, Euromedi equip ltd., West Harrow, UK). Hemoglobin was measured to the nearest 0.1 g/dL using a Hemocue machine (Mission Viejo, CA).

### Definitions

An uncomplicated pregnancy was defined as a pregnancy without the presence of AIDS-defining symptoms, hypertension, preeclampsia, polyhydramnios, abnormal fetal presentation, history of a cesarean section, hemorrhage, or repeated spontaneous abortions (>2). Placental parasitemia was defined as any plasmodial asexual form detected on a thick film. Maternal HIV infection was defined as a positive result on both rapid tests; women not reactive with the initial Serostrip HIV-1/2 test were considered HIV uninfected. Women whose serostatus could not be determined (i.e., those with discordant results on the two rapid tests and an indeterminate status with Western blot) were excluded from analysis. Newborns were classified as normal birth weight if they weighed >2,500 g, regardless of gestational age, and low birth weight if they weighed <2,500 g. Preterm delivery was defined as occurring at <37 weeks of gestation. Small for gestational age was defined as sex-specific birth weight <10th percentile for weight-for-gestational-age (*29*).

### Determining Infant HIV Status

An algorithm was developed to describe the perinatal HIV infection status of infants. Infants were classified as having acquired HIV infection perinatally (e.g., in utero or during labor and delivery) if they met the following conditions: 1) died and by the time of death had >2 consecutive positive HIV PCR tests, the first of which was at <4 months of age; 2) were lost to follow-up but had had >3 consecutive positive PCR results, the first of which was at <4 months of age; 3) remained alive with continued and consistent positive PCR results, the first of which was at <4 months of age. Infants were considered negative for perinatally acquired HIV if they had >3 PCR tests performed on them, and all tests were negative, with at least one of the negative tests at >4 months of age. Because infants had to be >4 months old to be classified as uninfected, those who died or were lost to follow-up before 4 months of age were excluded. Infants who acquired HIV at >5 months of age were considered to have acquired HIV postnatally and were included as nontransmitters from the perinatal perspective. Infants for whom we had insufficient PCR data to determine their status were classified as indeterminate and were excluded. Mothers of infected infants were classified as transmitters and those of uninfected infants as nontransmitters.

### Data Analysis and Statistical Methods

#### Univariate Analysis

Plasma virus levels load below the limit of quantification (400 copies/mL) were assigned a value of 200 copies/μL; plasma viral load results were then log_10_-transformed. We defined high-density placental parasitemia as >10,000 parasites/μL, which corresponded approximately to the uppermost quintile of parasite density. Univariate analyses were performed by using χ^2^ or Fisher exact tests (for cross-tabulations with an expected value in any cell <5) to compare proportions for categorical variables; *t* tests were used to compare normally distributed continuous variables. Relative risks (RRs) were computed with their 95% confidence interval (CI) to measure the strength of the associations between potential risk factors and perinatal MTCT.

### Multivariate Analysis

To evaluate the effect of placental malaria on perinatal MTCT, a Poisson log-linear model containing placental malaria and maternal viral load as primary predictor variables was constructed by using backward elimination; adjusted RRs were computed. An interaction term between placental malaria density and maternal viral load was significant (p = 0.02) and was retained in the model. Because women with and without placental malaria may have different risk factors for perinatal MTCT, we fit three separate multivariate models: all study women, women without placental malaria, and women with placental malaria. All tests were two-sided; p values <0.05 were considered significant. Analysis was done using STATA (StataCorp. 2001. Stata Statistical Software: Release 7.0 College Station, TX) and SAS (Version 8.0, SAS Institute, Cary, NC).

## Results

### Study Population

A total of 829 mother-infant pairs were enrolled; 317 (38.2%) infants with incomplete follow-up or indeterminate HIV status were excluded, leaving 512 mother-infant pairs. Included and excluded women did not differ in age, level of education, mean maternal viral load, mean CD4+ counts, malaria rates, high-density malaria rates, proportion of low birth order (i.e., gravida <2 versus >3), and rates of episiotomy or perineal tear. However, excluded women were more likely to have infants born with low birth weight (9.1% versus 5.5%, p = 0.04). The baseline characteristics of the 512 women included in the analysis are shown in Table 1. Among these 512 women, 128 (25.0%) had placental malaria and 353 (84.4%) had anemia (hemoglobin <11 g/dL).

**Table 1:** Characteristics of HIV-positive women and their newborns participating in perinatal HIV transmission study, Kisumu, western Kenya, 1996–2001^a^

Characteristic	All women (N = 512)^b^	
Maternal sociodemographic		
Luo ethnicity	86.5%	
Mean age (y) ± SD	22.4±4.4 (range 14–39)	
Mean gravidity ± SD	2.3±1.4 (range 1–9)	
Primigravid	35.9%	
Completed primary education (≥8 y)	68.0% (n = 510)	
No salaried employment	74.3%	
Married	78.4%	
History of fever and treatment for malaria		
History of fever previous week at screening	23.2% (n = 509)	
History of fever a fortnight before delivery	28.0% (n = 511)	
Treated with antimalarials in current pregnancy	30.9%	
Treated with chloroquine during current pregnancy	16.6%	
Axillary temperature ≥37.5°C at screening	2.9% (n = 455)	
Laboratory		
VDRL-positive	7.3% (n = 385)	
Hemoglobin <11 g/dL at screening	84.4% (n = 418)	
Hemoglobin <8 g/dL at screening	20.6% (n = 418)	
Mean maternal CD4+ count (% <200 cells/μL) 1 mo postpartum	629±334 (4.7%) (n = 464)	
Mean maternal log_10_ viral load at delivery (% below detection limit of 400 copies)	3.28±0.92 (33.0%) (n = 455)	
Peripheral parasitemia at screening	21.9% (n = 415)	
Peripheral parasitemia at delivery	19.7% (n = 497)	
Placental malaria	25.0%	
Delivery		
Episiotomy or perineal tear	36.4%	
Mean duration of rupture of membranes ± SD (% >4 hours)	2.7±6.2 (15.4%)	
Newborn		
Mean birth weight (% low birth weight)	3144±420 (5.5%)	
Prematurity (<37 wks completed gestation)	8.2%	
Maternal HIV transmitters	102 (19.9%)	

^a^VDRL, venereal disease research laboratory slide test. ^b^If characteristic not measured for all 512 women, n is given in parentheses.

### Correlates of Perinatal MTCT

Of 512 mothers, 102 (19.9%) transmitted HIV to their infants perinatally. High maternal viral load (>10,000 viral copies/μL) and low CD4+ count (<200 cells/μL) were significantly associated with perinatal MTCT (Table 2). When analyzed as continuous variables, transmitting mothers had higher geometric mean viral loads than nontransmitting mothers (7,083 versus 1,378 copies/μL; p<0.001) and low mean CD4+ count (511 versus 657 cells/μL; p<0.001). Other characteristics significantly associated with perinatal MTCT included episiotomy or perineal tear, low birth weight or small-for-gestational-age infants, and being of low birth order (primigravid or secundigravid).

**Table 2:** Risk factors associated with perinatal HIV infection by maternal viral, immunologic, obstetric, and other factors (univariate analysis), western Kenya, 1996–2001

Variable	No. studied	No. infected (%)	Relative risk (95% confidence interval)	p
Viral load >10,000				
No	358	50 (14.0)		
Yes	97	40 (41.2)	3.0 (2.1 to 4.2)	<0.001
CD4+ cells <200				
No	442	74 (16.7)		
Yes	22	13 (59.1)	3.5 (2.4 to 5.3)	<0.001
Hemoglobin <8 g/dL at screening				
No	332	58 (17.5)		
Yes	86	22 (25.6)	1.5 (1.0 to 2.3)	0.09
3rd-trimester maternal parasitemia				
No	324	64 (19.8)		
Yes	91	15 (16.5)	0.8 (0.5 to 1.4)	0.48
Maternal parasitemia at delivery				
No	399	83 (20.8)		
Yes	98	15 (15.3)	0.7 (0.4 to 1.2)	0.22
Placental malaria				
No	384	84 (21.9)		
Yes	128	18 (14.1)	0.6 (0.4 to 1.0)	0.05
Ever been treated for tuberculosis				
No	496	98 (19.8)		
Yes	13	4 (30.8)	1.6 (0.7 to 3.6)	0.33
Treated with chloroquine during pregnancy				
No	427	88 (20.6)		
Yes	85	14 (16.5)	0.8 (0.5 to 1.3)	0.38
Treated for vaginal discharge				
No	477	93 (19.5)		
Yes	32	9 (28.1)	1.7 (0.8 to 2.6)	0.24
Hospitalized during current pregnancy				
No	472	95 (20.1)		
Yes	39	7 (18.0)	0.9 (0.4 to 1.8)	0.74
History of fever 2 wks before delivery				
No	368	70 (19.0)		
Yes	143	32 (22.4)	1.2 (0.8 to 1.7)	0.39
Episiotomy or perineal tear				
No	325	56 (17.2)		
Yes	186	46 (24.7)	1.4 (1.0 to 2.0)	0.04
Primi- or secundigravid				
No	190	26 (13.7)		
Yes	322	76 (23.6)	1.7 (1.1 to 2.5)	0.007
Low birth weight				
No	484	91 (18.8)		
Yes	28	11 (39.3)	2.1 (1.3 to 3.4)	0.008
Prematurity				
No	468	92 (19.7)		
Yes	42	10 (23.8)	1.2 (0.7 to 2.1)	0.52
Small for gestational age				
No	444	83 (18.7)		
Yes	66	19 (28.8)	1.5 (1.0 to 2.4)	0.06

Maternal peripheral parasitemia at delivery was not associated with MTCT; however, placental malaria was associated with a 40% reduction in the risk for perinatal MTCT (RR 0.6, 95% CI 0.4 to 1.0, p = 0.05) (Table 2). Compared to women without malaria (perinatal MTCT rate = 21.9%), women with placental parasitemia at lower densities (<10,000 parasites/μL) had a lower rate of perinatal MTCT (11.5%); this was not true for women with higher-density placental parasitemia (>10,000 parasites/μL, perinatal MTCT rate = 25.0%), who represented approximately one fifth of women with malaria. Among women with viral load below the detection level, perinatal MTCT occurred in none of 35 mothers with malaria, compared to 10 (8.7%) of 115 mothers without malaria (p = 0.12).

### Multivariate Analysis of Risk Factors for Perinatal MTCT

Among all study women, high maternal viral load and episiotomy or perineal tear were independent risk factors, and low-density (but not high-density) malaria was an independent protective factor for perinatal MTCT. Among women without microscopically detectable malaria, high maternal viral load, gravidity <2, and low birth weight in the newborn were significant risk factors or markers for perinatal MTCT. Among women with malaria (where perinatal MTCT was overall lower than in the malaria-negative women), high maternal viral load, episiotomy or perineal tear, and high-density placental infection were significant risk factors for perinatal MTCT (Table 3). In both the multivariate models for the entire population and women with malaria, significant interaction was found between viral load and placental malaria density.

**Table 3:** Multivariate analysis of risk factors for perinatal HIV transmission, western Kenya, 1996–2001

	Adjusted relative risks (ARR) for perinatal HIV transmission^a^					
	All women^b^, N = 454		Placental malaria–negative, n = 348		Placental malaria–positive^b^, n = 107	
	ARR (95% CI)	p	ARR (95% CI)	p	ARR (95% CI)	p
Log_10_ viral load	1.8 (1.6 to 2.1)	<0.001	1.7 (1.4 to 2.0)	<0.001	3.5 (2.5 to 4.8)	<0.001
Episiotomy or perineal tear	1.6 (1.2 to 2.1)	0.004	–		4.8 (2.3 to 9.7)	<0.001
Low birth weight	–		1.9 (1.1 to 3.2)	0.03	–	
Gravidity <3 versus >3	–		1.8 (1.2 to 2.8)	0.003	–	
Placental malaria status						
Negative	Reference^c^		N/A			
<10,000 parasites/μL	0.4 (0.2 to 0.6)^b^	<0.001	N/A		Reference	
>10,000 parasites/μL	0.7 (0.3 to 21.5)^b^	NS	N/A		2.0 (1.1 to 3.9)	0.04

^a^–, factor was not retained in the final model; CI, confidence interval; N/A, not applicable; NS, not significant. ^b^A significant interaction was found between viral load and placental malaria density (p = 0.02) in these analyses. The effect of this interaction on the relative risk for placental malaria is shown in Figures 1 and 2. All relative risks given in this table do not include this interaction but give a weighted average of the placental malaria effect at various levels of viral load. ^c^An alternative model, in which placental malaria was fit as a binary variable (positive or negative), showed that placental malaria was protective for perinatal HIV transmission (relative risk 0.4, 95% CI 0.3 to 0.7, p < 0.001). In that model, log_10_ viral load and episiotomy or perineal tear remain independent risk factors.

In further examination of the interaction between maternal viral load and placental malaria, we found no significant differences in the frequency of episiotomy or perineal tear, mean CD4+ count, mean maternal hemoglobin level, mean birth weight, and frequency of small-for-gestational-age neonates among women without malaria, those with low-density placental malaria, and those with high-density placental malaria. Women with placental malaria were of lower mean gravidity than women without malaria (2.1 vs. 2.4, p = 0.03). Geometric mean maternal viral load was slightly higher in women with low-density placental malaria (2,226 copies/μL) and was nearly twofold higher in women with high-density placental malaria (3,390 copies/μL) than the viral load in women without placental malaria (1,774 copies/μL); however, these differences were not significant (p = 0.14). Viral load was significantly higher among women with peripheral malaria parasitemia at delivery (2,979 copies/μL) than among women without (1,725 copies/μL, p = 0.03). As shown in Table 4, the geometric mean viral load was fivefold higher in transmitters than in nontransmitters. While in women without malaria the geometric mean viral load in transmitters was fourfold higher than in nontransmitters, among women with malaria, the geometric mean viral load was 25-fold higher in transmitters than in nontransmitters. Among transmitting women, those with malaria had an eightfold higher geometric mean viral load than those women without malaria.

**Table 4:** Association between maternal viral load and placental malaria among women who did and did not transmit HIV perinatally to their infants, western Kenya, 1996–2001

	Geometric mean HIV viral load^a^			
	Transmitters	Nontransmitters	p value^b^	
All women (N = 455)	7,083	1,378	<0.001	
Placental malaria–positive	41,217	1,675	<0.001	
Placental malaria–negative	5,402	1,286	<0.001	
p value	0.002	0.26		

^a^Viral load expressed as copies per microliter of plasma. ^b^p from *t* test.

**Figure 1:**
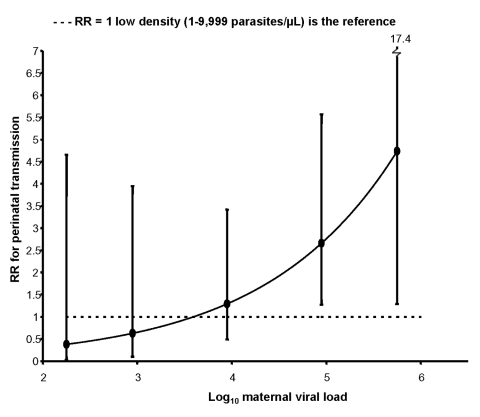
The effect of viral load and placental malaria density on risk for perinatal HIV transmission, western Kenya, 1996–2001. Women with low- (<10,000 parasites/μL, circles) and high- (>10,000 parasites/μL, squares) density placental malaria are compared with women without placental malaria (represented by the horizontal dashed line). RR, relative risk. Error bars refer to 95% confidence interval.

**Figure 2:**
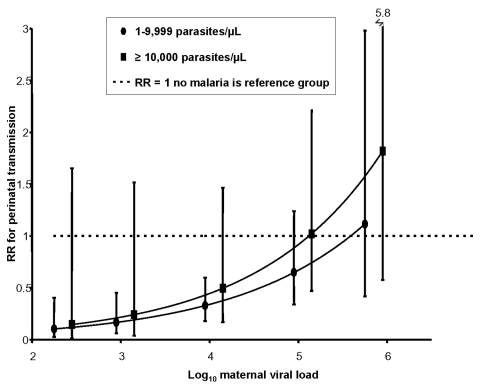
The effect of viral load and high-density placental malaria on risk for perinatal HIV transmission, western Kenya, 1996–2001. Women with high-density placental malaria (>10,000 parasites/μL) are compared to those with low-density placental malaria (<10,000 parasites/μL, represented by the horizontal dashed line). RR, relative risk. Error bars refer to 95% confidence interval.

These associations were further evaluated in models comparing the relative risk for perinatal transmission between the three groups with placental malaria at various levels of maternal viral load. Low-density placental parasitemia was associated with significant protection for perinatal MTCT at the lower viral load levels, but not at higher viral load levels (Figure 1). When women with low- and high-density parasitemia were compared (Figure 2), high-density placental parasitemia was associated with increased risk for perinatal MTCT but only at the higher viral load levels.

## Discussion

This evaluation of perinatal HIV transmission in a malarious area of western Kenya demonstrated that approximately 20% of infants born to HIV-infected mothers acquired HIV by 4 months of age, similar to rates reported in other sub-Saharan African settings (*30*–*32*). Consistent with other studies, we found that maternal viral load in peripheral blood at the time of delivery and having an episiotomy or perineal tear were risk factors for perinatal MTCT (*33**,**34*). Contrary to our expectations, we observed that women with placental malaria had lower rates of perinatal MTCT than women without malaria. However, women with high-density malaria had significantly higher rates of perinatal MTCT than parasitemic women with low-density malaria. In additional models, only low placental parasite density (1–9,999 parasites/μL of blood) was associated with protection from perinatal MTCT; high-density placental infection was either a risk factor for perinatal MTCT (in the model evaluating only women with malaria) or it was a neutral factor (in the model evaluating all women). Maternal viral load was slightly higher in women with high-density malaria, and, among transmitting women, mean viral load was eightfold higher if the woman had concurrent malaria, consistent with the suggestion that high-density malaria may be an important stimulus of viral replication (*10*–*12*).

Our study had some important limitations. First, only healthy women were screened for this study; no women with AIDS or any known underlying chronic illness were enrolled. Although this eliminated potential conditions that could have complicated the analysis (e.g., a higher likelihood of additional concurrent infections), it restricted our study population to the “healthiest” women with HIV and likely resulted in an underestimate of the overall rate of perinatal MTCT in our study area. Some infants categorized as having acquired HIV through perinatal transmission may have actually acquired it through early breastfeeding transmission. However, including these infants would likely bias the results toward underestimating the magnitude of the observed risks. Our measurement of malaria was limited to microscopy examination of placental smears and could have been inexact; those with no evidence of malaria may have had very low-density infection and may have been misclassified as having no malaria. Such misclassification would be expected to bias our findings toward the null hypothesis. Because placental malaria can cause an inflammatory response in the placenta, the use of leukocyte count to calculate parasite density may have resulted in an underestimation. However, our cutoff of 10,000 parasites/μL for high-density placental parasitemia is essentially a relative measure based on the upper quintile of densities; therefore, we do not think that our estimation technique would have introduced any bias. Finally, in a study such as ours, loss to follow-up always has the potential to introduce bias. Approximately one third of infants enrolled in our study were lost to follow-up. However, as noted, these mothers and infants were generally similar to the study population included in our analysis, and we were unable to detect biases that would have affected our analysis.

Our observation of an association between low-density placental malaria and reduced perinatal MTCT has several possible explanations. First, over 16% of women reported self-treatment (typically for fever) with chloroquine during pregnancy. High-grade chloroquine resistance in this area is widespread, and its use is unlikely to clear placental infections but may reduce parasite densities. Chloroquine is known to have anti-HIV properties and to reduce HIV-1 replication and viral loads in adults (*35*,*36*) and as such could potentially reduce the risk for perinatal MTCT. Although we identified no differences in the proportion of women with or without malaria who used chloroquine, and no association was found between chloroquine usage and MTCT, women identified as having malaria may have used chloroquine more frequently and at higher doses (information that was not collected) than women without malaria. A second possibility is that some of the women classified as having placentas negative for malaria parasites may actually have had low-level chronic malaria associated with inflammation and increased HIV replication. In contrast, women classified as having low-density malaria may have had recent malaria with minimal inflammatory response and thus no increase in HIV replication. Unfortunately, blood films are not capable of detecting inflammatory responses.

A more likely explanation is that a balance exists in the uterine-placental-fetal environment among malaria-induced antigen stimulation, HIV viral replication, maternal host immune response to both malaria and HIV, and the likelihood of MTCT. Although high viral load has been shown to increase the risk for MTCT, no more than half of exposed infants, even at high maternal viral load, become infected with HIV-1. These data suggest that other systemic or placental factors must be important in preventing HIV-1 transmission. Recent studies suggest that selected cytokines and hormones potentially affect HIV-1 transplacental transmission and that both innate and acquired protection play a role in MTCT (*37*). First, malaria is known to induce disequilibrium in Th1 and Th2 cells, favoring the Th1 pathway (*38*–*40*). T-helper responses are known to control HIV replication; hence, inducing Th1 response in the placental compartment could lead to reduced HIV-1 replication (*41*,*42*). Indeed, a moderate increase was found in the Th1 cytokine interferon-γ response in the intervillous blood mononuclear cell responses of HIV-positive mothers with malaria as compared to HIV-positive mothers without malaria (*43*). Second, leukemia inhibitory factor induces a potent inhibition of HIV replication, and this cytokine is upregulated in placentas of women who do not transmit HIV (*44*). Malarial antigens may induce production of leukemia inhibitory factor that results in reduced rates of perinatal MTCT. Third, malarial antigen may result in altered chemokine production, which in turn can block chemokine receptors necessary for cellular HIV entry (*45*). Recent immunologic studies conducted in a group of women who participated in this cohort showed that macrophage inflammatory protein (MIP)-1β, a chemokine known to block the entry of HIV-1, was significantly elevated in the intervillous blood plasma of women with placental malaria irrespective of their HIV status. In women with concurrent HIV and placental malaria, the intervillous blood plasma levels of MIP-1β were significantly elevated compared to HIV-negative women with malaria and HIV-positive women without malaria (*46*). Thus, a complex balance of antigen stimulus and immune response may occur in the placenta, as demonstrated in recent studies focusing on the placental intervillous blood responses (*38*–*40*,*43*,*46*). In this scenario, active malaria might lead to one of the local immune responses described. This response may control the density of parasitemia and may also provide immune control that limits perinatal MTCT. For most women with malaria (approximately 80% in our study), this resultant balance leads to “protection” with controlled (low-density) malaria and reduced perinatal MTCT. For a minority of women with malaria (approximately 20% in our study), the balance is tilted to inadequate control of malaria (i.e., higher density of malaria parasites with concomitant increased antigen stimulation of viral replication and higher viral load) and higher rates of perinatal MTCT. This finding is consistent with our observation that women with high-density placental malaria do have higher rates of perinatal MTCT than women with low-density placental malaria, but that they require much higher viral loads to achieve this transmission. Our findings suggest complex relationships between malaria, viral load, and perinatal MTCT. These findings will need to be confirmed and expanded upon in further research in different settings.

The finding of reduced perinatal MTCT in the presence of low-density, but not high-density, placental malaria does not suggest altering existing recommendations for the use of intermittent preventive antimalarial treatment in pregnant women in malarious areas of Africa (*47*). For most dually (malaria and HIV) infected women, the overall outcome would have modest benefit in reducing perinatal MTCT, but for the minority with insufficiently controlled infections, perinatal MTCT would be increased. At this point, we cannot differentiate in advance which women will end up in which group. The important benefit of antimalarial treatment may be to reduce the likelihood of women having high-density placental malaria and in also helping reduce the other known adverse effects of malaria during pregnancy, including anemia, low birth weight, and prematurity (*1*).

As noted earlier, this study was carried out in Kenya in a changing environment of perinatal HIV and malaria prevention and has been transformed into a program delivering short-course antenatal antiretroviral therapy to HIV-infected women and a system to support providing intermittent preventive antimalarial treatment during pregnancy according to the newly adopted national policy (*48*). As more countries adopt policies of antiretroviral and antimalarial interventions in pregnancy, the interrelationship and ultimate benefit of these two interventions need to continue to be evaluated.
